# Histopathologic and Immunohistochemical Evaluation of Induced Lesions, Tissue Tropism and Host Responses following Experimental Infection of Egyptian Rousette Bats (*Rousettus aegyptiacus*) with the Zoonotic Paramyxovirus, Sosuga Virus

**DOI:** 10.3390/v14061278

**Published:** 2022-06-12

**Authors:** Shannon G. M. Kirejczyk, Brian R. Amman, Amy J. Schuh, Tara K. Sealy, César G. Albariño, Jian Zhang, Corrie C. Brown, Jonathan S. Towner

**Affiliations:** 1 Department of Pathology, College of Veterinary Medicine, University of Georgia, Athens, GA 30602, USA; skirejc@gmail.com (S.G.M.K.); juliazh@uga.edu (J.Z.); corbrown@uga.edu (C.C.B.); 2 Viral Special Pathogens Branch, Centers for Disease Control and Prevention, Atlanta, GA 30333, USA; cxx1@cdc.gov (B.R.A.); wuc2@cdc.gov (A.J.S.); tss3@cdc.gov (T.K.S.); bwu4@cdc.gov (C.G.A.); 3 Division of Pathology, Emory National Primate Research Center, Emory University, Atlanta, GA 30329, USA

**Keywords:** Sosuga virus, zoonoses, paramyxovirus, *Rousettus aegyptiacus*, Egyptian rousette bat, natural reservoir, histopathology, immunohistochemistry

## Abstract

Ecological and experimental infection studies have identified Egyptian rousette bats (ERBs; *Rousettus aegyptiacus*: family Pteropodidae) as a reservoir host for the zoonotic rubula-like paramyxovirus Sosuga virus (SOSV). A serial sacrifice study of colony-bred ERBs inoculated with wild-type, recombinant SOSV identified small intestines and salivary gland as major sites of viral replication. In the current study, archived formalin-fixed paraffin-embedded (FFPE) tissues from the serial sacrifice study were analyzed in depth—histologically and immunohistochemically, for SOSV, mononuclear phagocytes and T cells. Histopathologic lesion scores increased over time and viral antigen persisted in a subset of tissues, indicating ongoing host responses and underscoring the possibility of chronic infection. Despite the presence of SOSV NP antigen and villus ulcerations in the small intestines, there were only mild increases in mononuclear phagocytes and T cells, a host response aligned with disease tolerance. In contrast, there was a statistically significant, robust and targeted mononuclear phagocyte cell responses in the salivary glands at 21 DPI, where viral antigen was sparse. These findings may have broader implications for chiropteran–paramyxovirus interactions, as bats are hypothesized to be the ancestral hosts of this diverse virus family and for ERB immunology in general, as this species is also the reservoir host for the marburgviruses Marburg virus (MARV) and Ravn virus (RAVV) (family *Filoviridae*).

## 1. Introduction

Bats (Order: Chiroptera) are drawing increased attention as natural reservoirs for viruses of public health importance, many of which are fatal for humans but cause little to no clinical disease in their bat hosts. These viruses include lyssaviruses such as rabies virus (family *Rhabdoviridae*), Hendra and Nipah viruses (NiV; family *Paramyxoviridae*), and the marburgviruses MARV and RAVV (family *Filoviridae*) [1,2,3,4,5,6,7,8,9,10,11]. Bats are hypothesized to be ancestral reservoirs of paramyxoviruses and coronaviruses, two virus families known for promiscuity among hosts [12,13,14,15]. Members of the family Paramyxoviridae comprise a diverse group of single-stranded, negative-sense RNA viruses and account for 1254 (9.0%) of the 13,973 publicly available genomic sequences derived from bats; 47 (3.7%) of these bat-derived paramyxovirus sequences are full length [16,17].

Within the family *Paramyxoviridae*, the subfamily *Rubulavirinae* comprises a diverse group of viruses infecting a variety of mammalian hosts, including humans, nonhuman primates, pigs, dogs and bats. In 2012, a novel rubula-like paramyxovirus, later named Sosuga virus (SOSV), was identified when a wildlife biologist became ill after performing field work on bats and rodents in South Sudan and Uganda, hence the name (South Sudan Uganda) [18]. Retrospective quantitative reverse transcriptase polymerase chain reaction (qRT-PCR) testing of pooled bat liver and spleen samples collected from bats captured at the fieldwork sites revealed that Egyptian rousette bats (ERBs) were the likely source of SOSV spillover [5]. The ERB is widely distributed throughout Africa, small parts of the Middle East, the Eastern Mediterranean and the Indian subcontinent. In Equatorial Africa, ERBs live in large, dense colonies, sometimes in excess of 100,000 individuals [10]. Ecological, epidemiological and experimental infection studies have identified ERBs as the primary source of marburgvirus spillover to primates, including humans [6,7,10,19,20,21]. ERBs have been associated with a number of novel viruses, including diverse, yet unnamed rubulaviruses and Bukakata orbivirus, two paramyxoviruses related to the henipaviruses, Achimota viruses 1 and 2 and Kasokero virus (KASV; family *Nairoviridae*), which have known zoonotic potential [22,23,24,25,26].

Experimental infection studies in colony-raised ERBs, using recombinant SOSV confirmed that this bat species is susceptible to infection, as evidenced by viremia, viral replication in multiple tissues, seroconversion and shedding in the feces, saliva and urine, all in the absence of overt clinical disease [27]. Tissue viral loads were highest in the small intestines, viral shedding events were most frequent in the feces and a subset of SOSV-infected bats had small intestinal lesions. Acute inflammation of the parotid salivary gland ducts was an unusual lesion in one 6 DPI bat that also had a high viral load in the salivary gland tissue and shedding into the oral secretions prior to necropsy [27]. These microscopic observations prompted a more in-depth investigation into the small intestines and salivary glands as target tissues, sources of shedding into the environment, and potential sites of localized immune responses to SOSV infection. Therefore, the goals of the current study were to describe and score the spectrum of histopathologic lesions in the small intestines and salivary glands to determine the tissue and cellular tropism of SOSV using immunohistochemical methods targeting tissues with high viral loads from the serial sacrifice study (small and large intestines, salivary gland, spleen and lymph nodes), and to characterize and quantify immune cell populations in the small intestines and salivary glands lesions using immunohistochemistry and whole-slide digital imaging analysis.

## 2. Materials and Methods

### 2.1. Tissue Samples

The ERB tissues used in the present study were obtained during a previously published serial sacrifice study performed under biosafety level 4 (BSL-4) conditions in accordance with safety regulations at the Centers for Disease Control and Prevention (CDC, Atlanta, GA, USA) [27]. The experimental infections were conducted with approval from the CDC Institutional Animal Care and Use Committee (IACUC protocol 2874TOWBATC), and in accordance with the animal welfare guidelines set forth in the Guide for the Care and Use of Laboratory Animals (Committee for the Update of the Guide for the Care and Use of Laboratory Animals, National Research Council of the National Academies 2011). Husbandry, housing conditions and diet for the duration of the experiment were previously described [27]. Briefly, food consisted of a variety of fresh fruit dusted with a protein vitamin supplement (Lubee Bat Conservancy, Gainesville, FL, USA). Water was provided ad libitum. The SOSV isolate was a recombinant virus genetically identical to that obtained from the index human case (GenBank ID: KF774436.1) [18]. Bats were euthanized under anesthesia via cardiac exsanguination at days 3, 6, 9 and 21 post-inoculation (DPI). Three control bats were mock-inoculated and euthanized at 21 DPI. Following tissue collection, the bat carcasses were immersed in 10% neutral buffered formalin (10% NBF) for a minimum of 7 days, with a complete change in formalin after day 3. Tissues were collected for histology within 3–4 weeks. 

### 2.2. Histology 

Representative sections from the following formalin-fixed tissues were prepared from bats at all time points and controls for histopathology: brain, heart, lung, trachea, thymus, liver, stomach, at least 13 cross-sections of small intestine (duodenum with pancreas, jejunum, and ileum), large intestine, spleen, kidney, adrenal gland, salivary gland, submandibular lymph node, axillary lymph node, skin from the inoculation site, skin from the chest region and pectoral muscle. Tissue sections were routinely processed, embedded in paraffin, sectioned at 4–5 µm thickness, mounted on glass slides and stained with hematoxylin and eosin (H&E). 

### 2.3. Immunohistochemistry (IHC)

Immunohistochemistry was performed on formalin-fixed, paraffin-embedded (FFPE) sections of small intestine, large intestine, spleen, axillary lymph node and salivary gland using a purified rabbit monoclonal antibody targeting the SOSV nucleoprotein (NP) (final dilution 1:2000; GenScript, Piscataway, NJ, USA). Tissue sections were heated at 60 °C and deparaffinized by washing in Hemo-De^®^ (Fisher Scientific, Pittsburgh, PA, USA). Antigen retrieval was achieved by applying an unmasking preheat solution (1 × Antigen Unmasking solution; Vector Laboratories, Burlingame, CA, USA) and microwaved at power level 10 for 2 min, followed by an additional 20 min of heating at power level 1. Slides were washed and then treated with a 1:10 dilution of Power Block Universal Blocking Reagent 10X (BioGenex Laboratories, San Ramon, CA, USA). The anti-SOSV NP antibody was applied and slides were incubated overnight at 4 °C. Primary Antibody Enhancer (Thermo Scientific, Waltham, MA, USA) was applied the following day to each slide for 20 min at room temperature. An alkaline phosphatase-based polymer detection system (Thermo Scientific, Waltham, MA, USA) with Fast Red chromogen was used. The slides were counterstained with hematoxylin (Leica, Richmond, IL, USA) and cover slipped with Permount for a permanent record. A formalin-fixed paraffin-embedded cell pellet created from a SOSV-infected Vero cell monolayer was used as a positive control. Tissues from the three non-infected ERBs were used as negative controls.

Immunohistochemical stains for ionized calcium-binding adapter molecule 1 (Iba1), a pan-macrophage and dendritic cell marker, and CD3, a T cell marker, were performed on sections of small intestine and salivary gland using an automated stainer (Nemesis 3600, Biocare Medical, Concord, CA, USA) at the University of Georgia Histology Laboratory. For the Iba1 protocol, a rabbit polyclonal antibody directed against Iba1 (1:8000 final dilution; Wako, Richmond, VA, USA.; catalog # 019-19741) was incubated on the tissue for 60 min. Antigen retrieval was performed using Antigen Retrieval Citra Solution 10X (BioGenex) at a 1:10 dilution for 15 min at 110 °C. For the CD3 protocol, a rabbit polyclonal antibody targeting the CD3 epsilon subunit (1:1000 final dilution; Dako, Carpinteria, CA, USA.; catalog # A0452) was incubated on the tissue for 60 min. For the cleaved caspase 3 protocol, a rabbit polyclonal antibody directed against activated (cleaved) caspase 3 (1:8000 final dilution; Biocare Medical, Concord, CA, USA.; catalog # CP229B) was incubated on the tissue for 60 min. Antigen retrieval was performed using Antigen Retrieval Citra Solution 10X (BioGenex) at a 1:10 dilution for 15 min at 110 °C. A biotinylated rabbit secondary antibody (Vector Laboratories, Burlingame, CA, USA) was used to detect the antibody targets, and the immunoreactions were visualized using a 3,3-diaminobenzidine substrate (DAB; Dako, Santa Clara, CA, USA) for 12 min. The slides were counterstained with hematoxylin and cover slipped. Positive control tissues included canine brain and lymph node for Iba1 and CD3, respectively. Each slide also contained internal controls (various lymphoid tissues). Immunostains were not performed on the salivary gland tissue of one bat (289953) due to tissue limitations.

### 2.4. Histopathological Lesion Scoring 

Histopathological lesions associated with SOSV infection in the small intestines and salivary glands were scored. Semi-quantitative scoring systems were designed and applied to the lesions in each tissue based on principles previously described in order to fully capture the spectrum of lesions observed and to allow for comparison between individual bats and time points [28]. At least 13 sections of small intestine (average = 18.4; range = 13–28) from each bat were reviewed in an unblinded manner to determine lesions of interest in SOSV-infected bats. Sections were then reviewed in a blinded manner without knowledge of time point or infection status and assigned a score for each of the lesions observed: villus epithelial discontinuity or ulceration (0 = absent, 1 = focal, 2 = multifocal, 3 = frequent); accumulation of eosinophilic fluid in the lamina propria (0 = absent, 1 = minimal to mild, 2 = moderate, 3 = marked); hypercellularity of the lamina propria (0 = none, 1 = minimal to mild, 2 = moderate, 3 = marked); epithelial cell apoptosis and/or tingible body macrophages in the lamina propria (0 = absent, 1 = rare, 2 = multifocal, 3 = frequent); and villus fusion (0 = absent, 1 = present). A composite score was generated for each bat by adding the individual criterion scores. The percentage of small intestinal sections affected for each bat was calculated by dividing the number of affected sections by the total number of sections examined. 

For the salivary glands, sections were reviewed in a blinded manner without knowledge of time point or infection status and assigned a score for each of the lesions observed: sialodochitis (0 = absent, 1 = minimal to mild, 2 = moderate, 3 = marked); sialadenitis (0 = absent, 1 = minimal to mild, 2 = moderate, 3 = marked); duct epithelial cell apoptosis (0 = absent, 1 = rare, 2 = multifocal, 3 = frequent); and epithelial syncytia (0 = absent, 1 = present). A composite score was generated for each bat by adding the individual criterion scores. 

### 2.5. SOSV Immunohistochemical Scoring

Tissues subjected to the anti-SOSV NP immunohistochemical procedure were scored as follows: - = no staining; + = <1 cell per 400× field; and ++ = >2 cells per 400× field. Immunostaining on intestinal tissue from bat 283936 (3 DPI) and salivary gland tissue from bats 290040 (21 DPI) and 290631 (control) was not performed due to tissue limitations.

### 2.6. Whole-Slide Digital Image Analysis (CD3 and Iba1 Staining Quantifications)

Tissues subjected to immunostaining for CD3 and Iba-1-immunostained scanned at 200× magnification using a whole-slide scanner (Aperio ScanScope XT, Aperio Technologies, Inc., Vista, CA, USA) and imported into a freely available open-source bioimage analysis software (QuPath version 0.3.0, Queen’s University Belfast, Belfast, Northern Ireland, UK) [29]. A region of interest (ROI) was manually generated around each of the small intestine and salivary gland sections. Immunolabeled cells were characterized by DAB binding and hematoxylin counterstaining. Labeled cells were segmented from negative cells and background staining by creating a threshold for each slide (DAB threshold range = 0.14–0.56) and using the other default parameters in the Create Threshold tool (gaussian sigma = 1.5 um, background radium = 15 um, cell detection threshold = 0.1, and detection object diameter = 25 pixels). False positives based on size and morphology of the cells were removed, as was background staining and artifact. 

The Fast Cell Counts tool was applied to each ROI to generate a cell count for CD3. For CD3, cell counts were normalized by dividing the total cell count by the area for each ROI in microns squared to obtain the number of cells per micron squared. A manual assessment of immunopositive cell morphology was performed for each slide as a means of quality control. For Iba1, % pixel positivity was obtained for each ROI using the Create Threshold tool, since the morphology (numerous cell processes) and occasional dense clustering of immunolabeled cells created difficulty in establishing accurate cell counts in some ROIs. 

### 2.7. Statistical Analyses

Statistical analyses were performed in Prism 9.3.1 (GraphPad Software, San Diego, CA, USA). For the histologic scores and the quantified Iba1 and CD3+ immunostaining, values at each time point (*n* = 3 per time point) were compared to those of the control bats using Kruskal–Wallis’ one-way analysis of variance (ANOVA) test followed by Dunnett’s multiple comparison test if the ANOVA demonstrated significant differences between groups (*p* < 0.05). Correlations between histologic scores and time points were analyzed using a simple linear regression. 

## 3. Results

### 3.1. Histopathology

Representative histologic sections of brain, heart, lung, trachea, thymus, liver, stomach, large intestine, spleen, kidney, adrenal gland, submandibular lymph node, axillary lymph node, pectoral muscle and skin from the inoculation site, wing and chest region were the same for SOSV-infected bats as the controls, with the following few exceptions. Four SOSV-infected bats (*n* = 1 at 3, 6, 9 and 21 DPI) had minimal to mild numbers of perivascular inflammatory cells in the lungs, one bat (6 DPI) had hyperplasia of the bronchus-associated lymphoid tissue (BALT) in the lungs, and one bat (3 DPI) had mild lymphohistiocytic interstitial nephritis.

#### 3.1.1. Small Intestines

**Figure 1:**
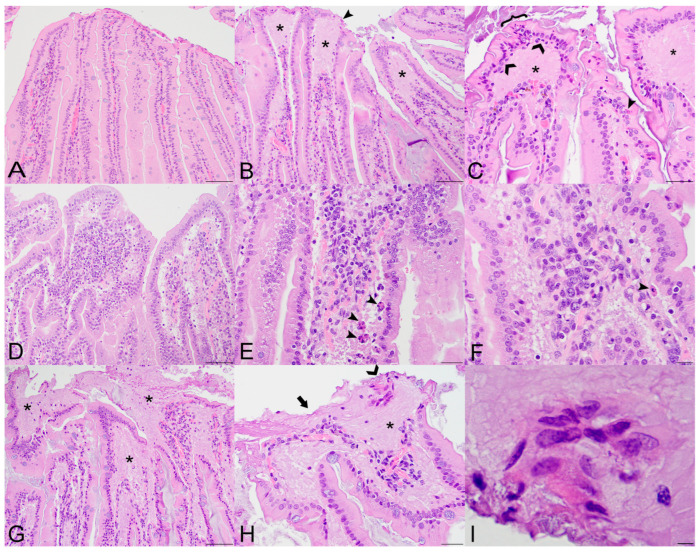
Histopathologic lesions in the small intestines of Sosuga virus (SOSV)-infected Egyptian rousette bats euthanized at 9 and 21 days post-inoculation (DPI). (**A**) Jejunum, control bat 290195. The intestinal villi are tall and slender with thin lamina propria cores. HE stain; original magnification = 200×; bar = 50 µm. (**B**) Jejunum, 9 DPI bat 287756. The villus tips are swollen with an accumulation of a paucicellular, eosinophilic fluid (*) that elevates the epithelium and compresses the lamina propria core. A villus tip is focally ulcerated (arrowhead). HE stain; original magnification = 200×; bar = 50 µm. (**C**) Jejunum, 9 DPI bat 287756. Enterocytes lining villus tips show a range of degenerative changes, including piling of nuclei, loss of cell borders and nuclear polarity ({) and nuclear fragmentation (closed arrowhead). Several mononuclear cells are present just beneath the epithelium (open arrowheads) and the lamina propria is expanded by eosinophilic fluid (*). HE stain; original magnification = 400×; bar = 20 µm. (**D**) Jejunum, 9 DPI bat 287014. The villi are multifocally fused and the lamina propria is congested and mildly hypercellular. HE stain; original magnification = 200×; bar = 50 µm. (**E**) Jejunum, 9 DPI bat 287014. The hypercellular lamina propria contains numerous tingible body macrophages beneath the epithelium (arrowheads). Inset: higher magnification of a tingible body macrophage from another but similarly affected villus showing phagocytosed cellular and nuclear debris. HE stains; original magnifications = 400×; bar = 20 µm. (**F)** Jejunum, 9 DPI bat 287014. Higher magnification image of the same bat demonstrating a congested and mildly hypercellular lamina propria containing a mixed inflammatory cell population composed of rounded to elongate macrophages with abundant cytoplasm, lymphocytes, and rare eosinophils and plasma cells. HE stain; original magnification = 600×; bar = 20 µm. (**G**) Duodenum, 21 DPI bat 287953. The villus tips are obscured by large ulcerations, accumulated eosinophilic fluid in the lamina propria (*) and multifocal villus fusion. Eosinophilic fluid spills into the lumen at the top of the image (*). HE stain; original magnification = 200×; bar = 50 µm. (**H**) Duodenum, 21 DPI bat 287953. The villus tip is folded over and has a focally extensive area of ulceration (arrow). At the top right of the image, a raft of syncytial epithelial cells (open arrowhead) is surrounded by eosinophilic fluid. HE stain; original magnification = 400×; bar = 20 µm. (**I**) Duodenum, 21 DPI bat 287953. High magnification image of the epithelial cell syncytium showing hypereosinophilic granular cytoplasm and karyolytic or pyknotic nuclei. HE stain; original magnification = 1000×; bar = 10 µm.

Lesions in SOSV-infected bats (n = 12; Figure 1) included villus epithelial ulcerations (8/12 bats; 75%), expansion of the lamina propria by eosinophilic fluid (8/12; 75%), lamina propria hypercellularity (10/12; 83%), increased apoptotic cells in the epithelium or just beneath the epithelium in the lamina propria (6/12; 50%), villus fusion (12/12; 100%) and epithelial syncytia (2/12; 17%). Lesions were randomly distributed throughout the representative intestinal segments (duodenum, jejunum, and ileum). Although the percentage of affected sections increased slightly over time from 17.8 % at 3 DPI to 39.4 % at 21 DPI, the association was not statistically significant. 

The first detectable tissue alteration at 3 DPI was the expansion of the lamina propria by a paucicellular eosinophilic fluid that elevated the epithelium and compressed the cellular core. Cellularity of the lamina propria varied between bats and was typically minimal to mild, although a few bats including one 6 DPI bat exhibited moderate hypercellularity with a predominantly mononuclear cell population that included numerous tingible body macrophages. Accumulation of fluid in the lamina propria resulted in expansion of the villus tips, and this was most evident in the 9 and 21 DPI bats (Figure 1B,C,G,H). Villus tip ulcerations were accompanied by degenerative changes in enterocytes adjacent to the epithelial defect, including loss of nuclear polarity, cytoplasmic hypereosinophilia and vacuolation and nuclear karyolysis or pyknosis (Figure 1C). Increased apoptotic enterocytes or subepithelial tingible body macrophages were most numerous in two of the 9 DPI bats (287014 and 287253; Figure 1E,F). At 21 DPI, villus ulceration was extensive, with spilling of fluid from the lamina propria into the gastrointestinal lumen (Figure 1G,H). Rare epithelial syncytia were observed within the lamina propria at the villus tips or within the gastrointestinal lumen (Figure 1I). Beginning with the 9 DPI bats, crypt epithelial cells exhibited the following changes indicative of a regenerative response: lengthening of the crypt, increased cytoplasmic basophilia and increased mitotic figures. Villus fusion was seen multifocally in all SOSV-infected bats and in one control bat that also had a mild to moderate multifocal eosinophilic and lymphocytic enteritis and mild hepatitis as background lesions. The remaining control bats had no lesions in the small intestines.

#### 3.1.2. Salivary Glands

**Figure 2:**
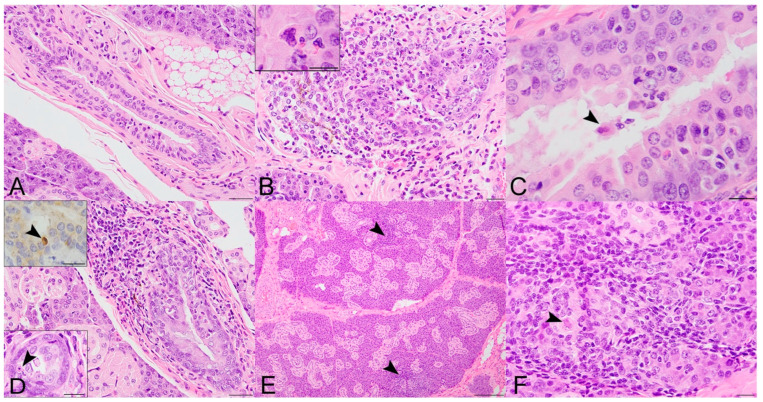
Histopathologic lesions in the salivary glands of SOSV-infected Egyptian rousette bats euthanized at 6, 9 and 21 DPI. (**A**) Salivary gland, control bat 290195. A normal interlobular duct is 1–3 cell layers thick and contains very low numbers of intraepithelial and peri-ductular resident immune cells. HE stain; original magnification = 400×; bar = 20 µm. (**B**) Salivary gland, 6 DPI bat 284899. Acute sialodochitis characterized by a robust infiltrate of mononuclear and granulocytic inflammatory cells that expands the duct wall and surrounding connective tissue. Granulocytic cells, mononuclear cells and cellular debris fill and partially occlude the duct lumen. Inset: The luminal aspect of a different, but similarly affected duct contains an epithelial syncytium and several granulocytes. HE stains; original magnifications = 400× (main image; bar = 20 µm) and 1000× (inset; bar = 10 µm). This bat was qRT-PCR positive for SOSV in the salivary gland at necropsy and had SOSV RNA in the oral swabs on days 5 and 6 PI. (**C**) Salivary gland, 9 DPI bat 287014. The duct is multifocally infiltrated by low numbers of mononuclear and granulocytic cells. The lumen contains low numbers of neutrophils and a binucleated cell with abundant granular cytoplasm (suspected syncytium) (arrowhead). HE stain; original magnification = 400×; bar = 20 µm. (**D**) Salivary gland, 21 DPI bat 289338. An interlobular duct is focally infiltrated and expanded by a mixture of mononuclear and granulocytic inflammatory cells. Bottom left inset: The wall of a smaller but similarly affected duct contains an apoptotic body that is shrunken and rounded with distinct cell borders and a pyknotic nucleus (arrowhead). HE stains; original magnifications = 400× (main image; bar = 20 µm) and 1000× (inset; bar = 10 µm). Top left inset: An apoptotic body in the wall of the same duct as the lower left inset has diffuse, moderate cytoplasmic immunoreactivity for cleaved caspase 3 (arrowhead). Immunohistochemical stain with 3-3′-Diaminobenzidine (DAB; brown) chromogen to visualize cleaved caspase 3 antigen and hematoxylin counterstain. Original magnification = 600×; bar = 20 µm. (**E**) Salivary gland, 21 DPI bat 290040. Low magnification image of salivary gland acinar tissue showing multiple foci of mononuclear cells which expand the interstitium and focally obscure the acini (arrowhead). HE stain; original magnification = 200×; bar = 50 µm. (**F**) Salivary gland, same 21 DPI bat as previous image. Higher magnification of one hypercellular focus showing interstitial expansion with numerous mononuclear inflammatory cells. One intralobular duct epithelial cell exhibits cytoplasmic swelling, peripheralization of nuclear material and accumulation of eosinophilic material within the nucleus suggestive of intranuclear inclusion material (arrowhead). HE stain; original magnification = 400×; bar = 20 µm.

The spectrum of salivary gland lesions in SOSV-infected ERBs (*n* = 12; Figure 2) included the presence of apoptotic cells within the interlobular duct epithelium (10/12; 83.3%), sialadenitis (7/12; 58.3%), sialodochitis (5/12 bats; 41.2%) and syncytial cells (2/12; 17%). Eleven of the 12 SOSV-infected bats (91.7%) had at least one of the aforementioned lesions and the only SOSV-infected bat without salivary gland lesions was a 3 DPI bat. 

A mixed inflammatory cell infiltrate targeting the interlobular ducts (sialodochitis) was first observed in a 6 DPI bat (284899), where it was widespread and associated with epithelial cell degeneration, necrosis and apoptosis and rare syncytia formation (Figure 2B). Sialodochitis was minimal to mild in the 9 and 21 DPI bats and it was occasionally associated with epithelial hyperplasia, piling of epithelial cell nuclei, loss of nuclear polarity and rare epithelial syncytia (Figure 2C,D). At 9 and 21 DPI, there were also scattered aggregates of mononuclear cells within the lobular interstitium and rare acinar cell degeneration (Figure 2E,F). Intraductal cells exhibiting microscopic features consistent with apoptosis (shrunken cells with rounded cytoplasmic borders and pyknotic and/or fragmented nuclei) were first observed within the interlobular duct epithelia at 3 DPI, more frequently in ducts with inflammatory cell infiltrates but also in the absence of overt inflammation on the HE stain. Apoptotic cells were most numerous in two 21 DPI bats including the supershedder bat who shed SOSV periodically into the saliva during the serial sacrifice study (bat 289953; Figure 2D bottom left inset). Intraductal apoptotic bodies exhibited moderate diffuse cytoplasmic immunopositivity for cleaved caspase 3 (Figure 2D top left inset). One control bat had a small focus of lymphocytic inflammation adjacent to a sialolith. The remaining control bat salivary glands were histologically unremarkable.

#### 3.1.3. Histopathological Lesion Scores

**Figure 3:**
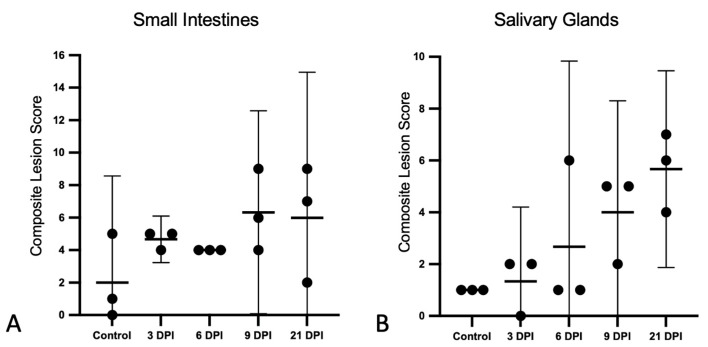
Histopathologic lesion scores in the small intestines and salivary glands of SOSV-infected Egyptian rousette bats. Individual groups are represented on the *x* axis and each dot represents the score for a single bat. Bars represent the mean and lines represent the ±95% confidence interval. Control bats were euthanized at 21 DPI. (**A**) Criteria used to generate a composite small intestine lesion score for each bat included: villus epithelial discontinuity or ulceration, accumulation of eosinophilic fluid in the lamina propria, hypercellularity of the lamina propria, epithelial cell apoptosis and/or tingible body macrophages in the lamina propria and villus fusion. (**B**) Salivary gland scores were positively correlated with time. Criteria used to generate a composite salivary gland lesion score for each bat included: sialodochitis, sialadenitis, duct epithelial cell apoptosis and epithelial syncytia.

The composite lesion scores for the small intestinal and salivary gland lesions are presented for each bat in Figure 3. For the small intestines, histologic lesion scores were similar for the 3 and 6 DPI. Compared to the controls, the mean scores were increased for the 9 and 21 DPI bats; however, these differences were not statically significant, and the scores did not correlate with time (Figure 3A). Salivary gland scores were positively correlated with time (r^2^ = 0.55; F = 16.51; *p* = 0.0013). 

### 3.2. Immunohistochemistry

#### 3.2.1. SOSV Tissue Tropism

**Figure 4:**
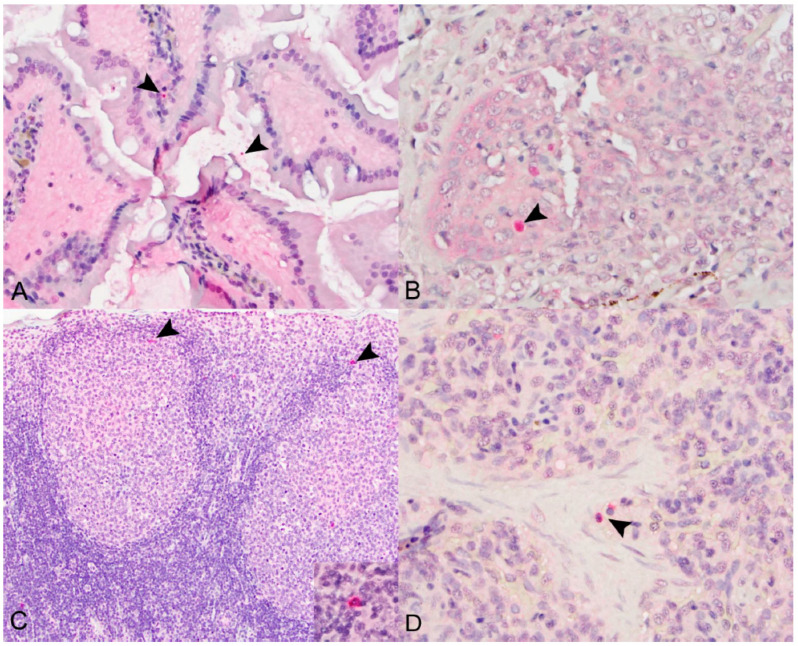
Immunohistochemical localization of SOSV nucleoprotein (NP) antigen in experimentally infected Egyptian rousette bats euthanized at 6, 9 or 21 DPI. (**A**) Small intestines, 21 DPI bat 289953. Granular, intracytoplasmic NP antigen is present within the villus lamina propria core and within the lumen (arrowheads). Immunohistochemical stain with Fast Red chromogen and hematoxylin counterstain; original magnification = 400×. (**B**) Salivary gland, 6 DPI bat 284899. Multifocal cytoplasmic immunolabeling for NP antigen within the wall of an inflamed interlobular salivary duct (arrowhead). Immunohistochemical stain with Fast Red chromogen and hematoxylin counterstain; original magnification = 400×. (**C**) Axillary lymph node, 6 DPI bat 284509. Viral nucleoprotein antigen (arrowheads) is present within the cytoplasm of histiocytic cells in the cortical germinal centers and in the paracortical regions (arrowheads and inset). Immunohistochemical stain with Fast Red chromogen and hematoxylin counterstain; original magnifications = 200× (main image) and 600× (inset). (**D**) Spleen, 9 DPI bat 287014. NP antigen (arrowheads) is present within the cytoplasm of few histiocytic cells in the red pulp. Immunohistochemical stain with Fast Red chromogen and hematoxylin counterstain; original magnification = 600×.

Immunohistochemical signal for SOSV NP antigen was detectable in one or more of the following tissues for 9 of the 12 (75%) SOSV-inoculated bats: small and large intestines, salivary gland, axillary lymph nodes and spleen (Table 1). In the intestines, viral antigen was localized to the cytoplasm of mononuclear cells in the lamina propria (*n* = 3; Figure 4A), within fluid in the gut lumen (*n* = 2) and in the gastrointestinal-associated lymphoid tissue (*n* = 2). In the salivary glands, viral antigen was observed within or surrounding the interlobular ducts (*n* = 2; Figure 4B). Of the tissues tested, viral antigen was most frequently detected in the axillary lymph nodes (*n* = 7); it was present in two of the three 21 DPI bats and in at least one bat at all other time points. In the lymph nodes, viral antigen was localized to the cytoplasm of histiocytic cells within the sinuses, germinal centers and paracortical regions (Figure 4C). In the spleen, antigen was present in the cytoplasm of macrophages within the red pulp (*n* = 4; Figure 4D). Viral antigen was not detected in tissues from the three control bats.

#### 3.2.2. Quantification and Spatial Analyses of Iba1- and CD3-Immunopositive Cell Populations

The subcellular localization for the Iba1 and CD3 antibodies was appropriate for both markers (cytoplasmic for Iba1 and membranous to cytoplasmic for CD3). 

##### Small Intestines

**Figure 5:**
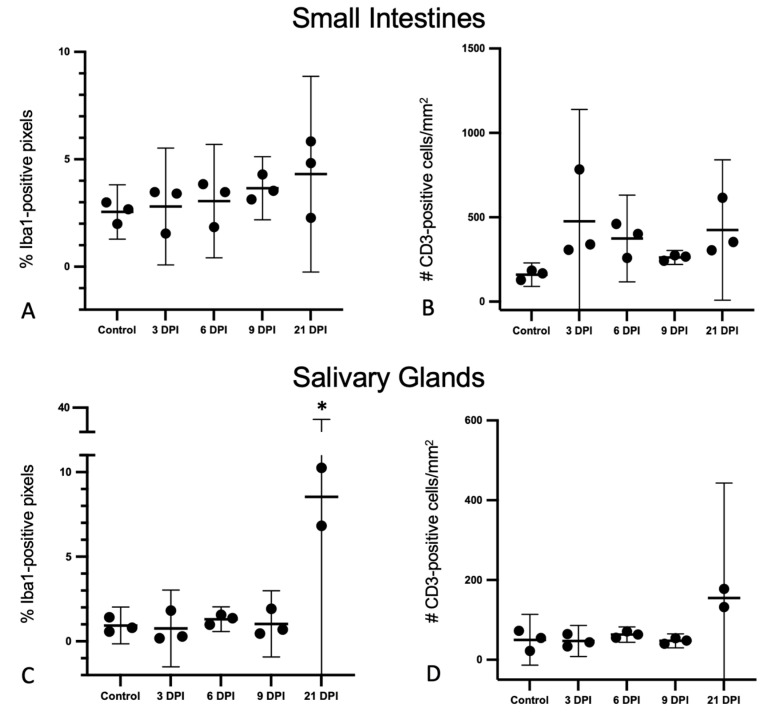
Iba1+ pixel percentages and CD3+ cell counts obtained by immunohistochemistry and whole-slide digital image analyses of the small intestines and salivary glands from SOSV-infected Egyptian rousette bats. Individual groups are represented on the x axis and each dot represents the score for a single bat. Bars represent the mean and lines represent the ±95% confidence interval. Control bats were euthanized at 21 DPI. (**A**) Percentage of Iba1-immunopositive pixels in the small intestines. (**B**) Number of CD3-immunopositive cells in the small intestines per mm^2^ tissue. (**C**) Percentage of Iba1-immunopositive pixels in the small intestines. The Iba1% positive pixel count was significantly increased in the salivary glands of SOSV-infected bats at 21 DPI relative to the controls (*). (**D**) Number of CD3-immunopositive cells in the small intestines per mm^2^ tissue.

**Figure 6:**
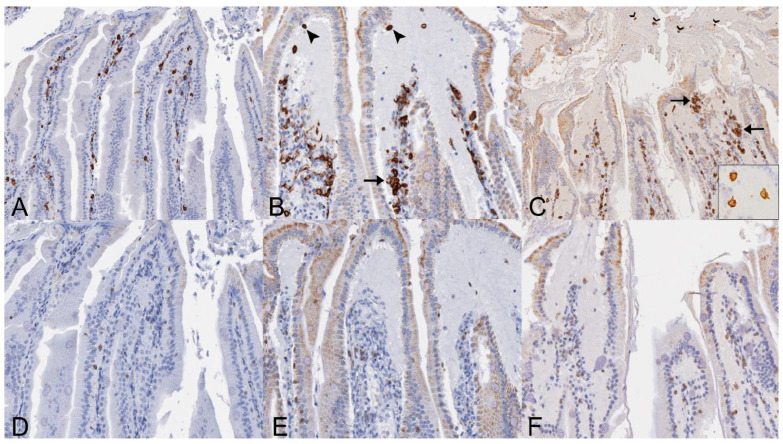
Distribution of Iba1- and CD3-immunolabeled cells in the small intestines of SOSV-infected Egyptian rousette bats euthanized at 9 and 21 DPI. (**A**) Iba1 immunohistochemistry (IHC), jejunum, control bat 290195. Iba1-immunolabeled cells (brown) are present in moderate numbers throughout the lamina propria core of the small intestinal villi. Original magnification = 200×. (**B**) Iba1 IHC, jejunum, 9 DPI bat 287756. Moderate numbers of Iba1-immunolabeled cells are present within the lamina propria core, occasionally in small clusters (arrow). Low numbers of Iba1+ cells with an amoeboid morphology are scattered within the lamina propria fluid and just beneath the epithelium at the villus tip (arrowheads). The small intestines of this bat had the highest viral load of all tissues tested during the serial sacrifice study. Original magnification = 200×. (**C**) Iba1 IHC, jejunum, 21 DPI bat 289953. The villus core and lamina propria contain moderate numbers of Iba1+ cells, occasionally clumped in small aggregates (arrows). Several villus tips have epithelial defects through which fluid spills from the lamina propria into the lumen. This luminal fluid contains several Iba1+ cells (open arrowheads). Inset: Higher magnification image demonstrating the amoeboid phenotype of macrophages present within the lamina propria fluid. This bat intermittently shed SOSV in the feces throughout the duration of the serial euthanasia study. Original magnifications = 100× (main image) and 600× (inset). (**D**) CD3 IHC, jejunum, control bat 290195. Low numbers of CD3+ cells (brown) are scattered throughout the epithelium. Rare CD3+ cells are present within the lamina propria core. Original magnification = 400×. (**E**) CD3 IHC, jejunum, 9 DPI bat 287756. Low numbers of CD3+ cells are scattered throughout the intestinal epithelium and occasionally present within the lamina propria fluid. Slightly increased CD3+ cells are present within the lamina propria core compared to the control. Original magnification = 400×. (**F**) CD3 IHC, jejunum, 21 DPI bat 289953. Scattered CD3+ cells are present just beneath the intestinal epithelium and within the lamina propria fluid. Slightly increased numbers of CD3+ cells are distributed throughout the lamina propria core compared to the control. Original magnification = 400×. All immunohistochemical stains were performed using 3-3′-Diaminobenzidine (DAB; brown) chromogen to visualize cellular antigen and hematoxylin counterstain.

There were no statistically significant changes in Iba1+ positive pixel of CD3+ cell counts in the small intestines. There was a very mild (1.69-fold) increase in the percentage of Iba1+ pixels when comparing SOSV-infected bats euthanized at 21 DPI (*n* = 3) to the controls (Figure 5A). In both infected and non-infected bats, Iba1+ cells were located predominantly within the lamina propria (Figure 6A–C). In SOSV-infected bats, Iba1+ cells occasionally formed small, dense clusters near the villus tips, particularly in fused villi (Figure 6B,C). In SOSV-infected bats, low numbers of Iba1+ cells were also present just beneath the epithelium, within the accumulated lamina propria fluid, and in the gut lumen, suggesting that these cells are responding to the tissue changes observed (Figure 6B,C). The morphology of Iba1+ cells within the lamina propria core was either mesenchymal (slender, elongate cells with long, branching cytoplasmic processes) or amoeboid (round to oval with large cell bodies and several, short cytoplasmic processes). Iba1+ cells within the lamina propria fluid of SOSV-infected bats often exhibited the amoeboid morphology (Figure 6C inset). 

CD3+ cell counts were highly variable between individual bats and although there were no statistically significant differences between groups, SOSV-infected bats tended to have higher CD3+ cell counts in the intestines compared to the control bats. There were also spatial differences in the distribution of CD3+ cells relative to controls (Figure 5B). In the control bats, the majority of CD3+ cells were distributed within the epithelium, with fewer immunopositive cells in the villus core (Figure 6D). In SOSV-infected bats, the lamina propria core contained slightly increased numbers of CD3+ cells. Immunopositive cells were also present in the accumulated lamina propria fluid in low numbers (Figure 6E,F). 

##### Salivary Glands

**Figure 7:**
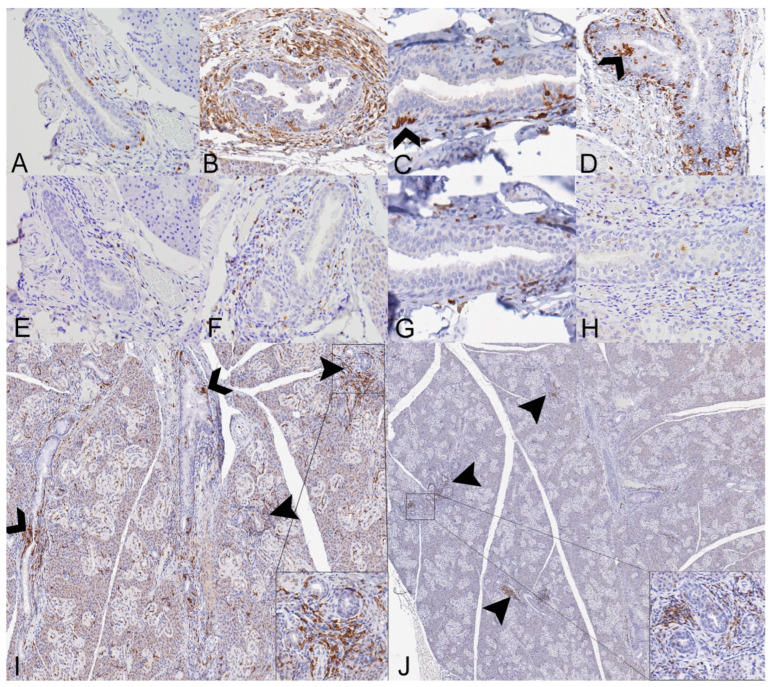
Distribution of Iba1- and CD3-immunolabeled cells in the salivary glands of SOSV-infected Egyptian rousette bats euthanized at 6, 9 and 21 DPI. (**A**) Iba1 immunohistochemistry (IHC), salivary gland, control bat 290195. Low numbers of Iba1+ cells (brown) are present within the salivary duct and surrounding connective tissue. Original magnification = 400× (**B**) Iba1 IHC, salivary gland, 6 DPI bat 284899. Numerous Iba1+ cells morphologically consistent with macrophages or dendritic cells infiltrate the salivary gland duct epithelium and expand the adjacent connective tissues. Original magnification = 400×. (**C**) Iba1 IHC, 9 DPI bat 287014. A small cluster of Iba1+ cells (open arrowhead) is present within a hyperplastic salivary duct. This bat had mild eosinophilic and neutrophilic sialodochitis, scattered apoptotic ductular epithelial cells and an epithelial syncytium. Original magnification = 350×. (**D**) Iba1 IHC 21 DPI bat 289338. Increased numbers of Iba1+ cells are distributed throughout a salivary duct, and multifocally in small clusters (open arrowhead). This bat had moderate eosinophilic sialodochitis, sialoadenitis and numerous apoptotic cells within interlobular ducts. Original magnification = 200×. (**E**) CD3 IHC, salivary gland, control bat 290195. Rare CD3+ cells (brown) are present within the salivary duct and surrounding connective tissue. Original magnification = 400×. (**F**) CD3 IHC, salivary gland, 6 DPI bat 284899. Moderate numbers of CD3+ cells infiltrate the periductular connective tissues. Original magnification = 400×. (**G**) CD3 IHC, 9 DPI bat 287014. Very low numbers of CD3+ cells surround an interlobular duct. Original magnification = 350×. (**H**) CD3 IHC 21 DPI bat 289338. Mild to moderate numbers of CD3+ cells surround and infiltrate an affected salivary duct. This bat had moderate eosinophilic sialodochitis, sialoadenitis and numerous apoptotic cells within interlobular ducts. Original magnification = 200x. (**I**) Iba1 IHC, 21 DPI bat 289338. Low magnification image demonstrating widespread Iba1+ immunolabeling and aggregation of these cells within interlobular ducts (open arrowheads). Iba1+ cells also form loose aggregates multifocally within the interstitium (closed arrowheads), where they separate, surround and interdigitate with acinar and intralobular duct epithelial cells (inset is higher magnification of boxed region). Original magnifications = 80× (main image) and 300× (inset). (**J**) CD3 IHC 21 DPI bat 289338. Low magnification image demonstrating loose aggregates of CD3+ cells distributed multifocally throughout the interstitium (arrowheads), where they separate, surround and infiltrate intralobular ducts (inset is higher magnification of boxed region). Original magnifications = 80× (main image) and 300× (inset). All immunohistochemical stains were performed using 3-3′-Diaminobenzidine (DAB; brown) chromogen to visualize cellular antigen and hematoxylin counterstain.

There were distinctive differences in the quantity and distribution of Iba1+ and CD3+ cells in the salivary glands of SOSV-infected bats compared to the controls, consistent with a controlled immune response to infection (Figure 7). There was a statistically significant 4-fold increase in the % of Iba1+ pixels at 21 DPI (*n* = 2) relative to the controls (Figure 5C; Dunnett’s multiple comparison test; *p* < 0.0001). There was a 3-fold increase in the number of CD3+ cells per mm^2^ at 21 DPI (*n* = 2) compared to the controls, but this was not statistically significant. There were no other statistically significant differences between groups. In the control bats, Iba1+ cells were distributed in low numbers within the duct epithelium and occasionally in small aggregates within the lobular interstitium (Figure 7A). There were no changes in the distribution of Iba1+ cells for the 3 DPI bats. The 6 DPI bat with sialodochitis exhibited the expansion of the periductal connective tissue with numerous Iba1+ cells and increased numbers of Iba1+ cells were also observed within the duct epithelium (Figure 7B). In the 9 and 21 DPI bats, Iba1+ cells were present in small clusters within the duct epithelium; these clusters were most numerous in the two 21 DPI bats analyzed (Figure 7C,D). Large aggregates of Iba1+ cells were also distributed throughout the salivary lobules, where they separated and surrounded acinar cells and intralobular ducts (Figure 7I). 

In the control bats, CD3+ cells were distributed in comparatively low numbers within the duct epithelium and occasionally in small aggregates within the lobular interstitium. The 3 DPI bats did not show any changes in the quantity or distribution of CD3+ cells. By 6 DPI, one bat with sialodochitis had increased numbers of CD3+ cells surrounding the inflamed ducts (Figure 7F). Slightly increased numbers of CD3+ cells were also present within and surrounding the affected ducts of 9 and 21 DPI bats (Figure 7G,H). There were increased interstitial CD3+ cell aggregates in the 21 DPI bats, which expanded the interstitium, surrounded and infiltrated intralobular ducts in moderate numbers and colocalized with Iba1+ cells (Figure 7J and inset). 

## 4. Discussion

Our histopathological and immunohistochemical findings corroborate the virological data previously reported. Histopathologically, SOSV infection was associated with villus tip ulcerations, lamina propria fluid accumulation and rare syncytia formation in the small intestines. The presence of viral antigen in the lamina propria and gut lumen demonstrates that shedding into the feces likely occurred via ulcerations at the villus tips or in shed enterocytes. The assumption that small intestines are a primary target tissue for SOSV replication and the likely principal source of virus shedding in experimentally infected ERBs was confirmed [27]. In the salivary glands, acute sialodochitis with epithelial cell apoptosis, necrosis, syncytia and intraepithelial viral antigen provides support for the salivary ducts as an additional site of SOSV replication and a source of shedding into the oral secretions. Average lesion scores in the small intestines and salivary glands increased over time, indicative of ongoing host responses to SOSV infection. Our findings in the tissues of SOSV-infected ERBs are aligned with the concept of disease tolerance, in which cellular and molecular damage control mechanisms limit the development of severe lesions, thereby avoiding clinical disease and promoting individual bat survival, virus persistence and asymptomatic infections [9,27,30,31,32,33,34,35,36]. 

Spatial, quantitative and temporal analyses of the Iba1+ and CD3+ immune cell populations revealed tissue-specific host responses following experimental SOSV infection. In the salivary glands, a targeted and robust immune cell response was evident at 21 DPI. In contrast, in the small intestines, immune cell counts increased only mildly despite high viral loads and ongoing enterocyte damage and loss. These results suggest that interactions between infected or damaged host cells and macrophages or T cells with distinct immunologic properties and the presence of soluble mediators may be important factors in limiting the development of severe, life-threatening disease despite disruption of the intestinal epithelial barrier. Intestinal macrophages have evolved to produce anti-inflammatory cytokines such as interleukin (IL)-10 and transforming growth factor (TGF) beta, thereby promoting the expansion of regulatory T cells and establishing tolerance to food antigens and commensal bacteria [37,38,39,40]. The amoeboid morphology of macrophages within the lamina propria fluid in SOSV-infected ERBs may provide some insight into their immunological phenotype, as alternatively activated (M2) macrophages are capable of transitioning from a mesenchymal to amoeboid migratory mode in response to a fluid extracellular milieu [41]. Furthermore, some M2 macrophage subtypes promote wound healing and tissue repair, which would be beneficial for SOSV-infected ERBs. Given that MARV has a documented tropism for mononuclear phagocytes in ERBs and fecal MARV shedding has also been reported, the involvement of this cell lineage in the ERB host response to SOSV infection is of interest [7,9,20]. 

Ongoing apoptosis of enterocytes and salivary duct epithelial cells, the detection of viral antigen in several tissues at 21 DPI and fecal shedding of one bat throughout the serial sacrifice study raise the potential for persistent or prolonged ERB–SOSV interactions. Chronic infections have been reported for a number of paramyxoviruses, including parainfluenza virus 3, measles virus and canine distemper virus, although the molecular mechanisms driving these prolonged host–virus infections remain unclear [42]. Recrudescence of SOSV may also be a concern in ERBs, as this phenomenon was the only plausible explanation for Nipah virus shedding in a captive closed colony of Malaysian flying foxes (*Pteropus vampyrus*) following the seroconversion of two naïve contact bats [43]. Persistent antigen in the axillary lymph nodes of SOSV-infected ERBs may serve as a driver of the adaptive immune response [44]. While antibody-mediated virus neutralization does not contribute significantly to the control and clearance of SOSV, anti-SOSV antibodies may serve other functions such as opsonization and phagocytosis or antibody-dependent cellular cytotoxicity [45]. 

## 5. Conclusions

The potential for persistent SOSV infection and a dampened or anergic immune response in the small intestines may have broader implications for host–paramyxovirus dynamics in chiropterans, a heterogenous group of mammals who are hypothesized to be the ancestral hosts of this diverse virus family. We identified the small intestines and salivary glands as primary target tissues for SOSV in ERBs, and described the disparate host responses in these tissues, which are likely a function of the anatomy, immunophenotypes of tissue resident immune cell populations and tissue-specific intrinsic repair and renewal capacities [36]. Despite histologic evidence of both direct and indirect tissue damage, the lesions were overall limited in scope and severity, and the lack of clinical disease supports the concept of disease tolerance for this model. In both tissues, immune responses were driven at least in part by mononuclear phagocytes and CD3+ cells. In the small intestines, there was direct damage to the villi, and subsequent immunopathology was likely limited by the intrinsic hypo-responsiveness of the resident immune cell populations and the high repair capacity of this tissue. The same or related mechanisms may have contributed to substantial shedding from this tissue. While there was evidence of immunopathology in the salivary glands, there was variability between individual bats, and overall, the lesions, similar to those of the intestines, were mild to moderate without any major changes to tissue structure or detectable losses of function. The robust inflammatory cell infiltrates at 21 DPI in the salivary glands suggests the consistent recruitment of immune cells from the circulation, and possibly the local expansion of resident immune cells in this tissue, which supports the release of inflammatory mediators. However, the clinical implications of damage to this tissue would be minor compared to those of the small intestines, and so the development of salivary gland lesions may not be as critical in terms of host fitness [36]. Additional tissue-based studies that preserve the spatial relationships between immune cells and infected host cells will be necessary to further resolve the induction of pro- and/or anti-inflammatory pathways in target tissues and to explore the anatomical and physiological features which have made this bat species a suitable natural reservoir for Marburg virus, Ravn virus and Kasokero virus.

## Figures and Tables

**Table 1:** Distribution of Sosuga virus nucleoprotein antigen in selected tissues from SOSV-infected Egyptian rousette bats euthanized at 3, 6, 9 and 21 DPI.

DPI	Bat ID	Small Intestines	Salivary Gland	AxLN	Spleen	Large Intestinal GALT
3	283936	NP	-	+	-	NP
	284049	-	-	+	-	-
	284459	-	-	-	-	-
6	284509	-	-	++	-	-
	284899	+	+	+	++	-
	285146	-	-	-	-	-
9	287014	-	-	-	-	-
	287253	-	-	-	+	-
	287756	+	-	+	+	+
21	289338	-	+	-	-	-
	289953 *	+	-	+	+	+
	290040	-	-	+	-	-
Control	290195	-	-	NP	NP	-
	290494	-	-	-	-	-
	290631	-	-	-	-	-

Abbreviations: DPI = days post-inoculation, AxLN = axillary lymph node, GALT = large intestinal gastrointestinal-associated lymphoid tissue, NP = not performed due to tissue limitations. IHC Scores: - = no staining, + = rare staining (<1 cell per 400× field), ++ = moderate staining (>2 cells per 400× field). * Indicates the supershedder bat identified during the serial euthanasia study [27].
